# Dietary Triacylglycerols with Palmitic Acid in the sn-2 Position Modulate Levels of N-Acylethanolamides in Rat Tissues

**DOI:** 10.1371/journal.pone.0120424

**Published:** 2015-03-16

**Authors:** Gianfranca Carta, Elisabetta Murru, Sara Lisai, Annarita Sirigu, Antonio Piras, Maria Collu, Barbara Batetta, Luisa Gambelli, Sebastiano Banni

**Affiliations:** 1 Dipartimento Scienze Biomediche, Università di Cagliari, Cagliari Italy; 2 Loders Croklaan Europe, Wormerveer, The Netherlands; GDC, GERMANY

## Abstract

**Background:**

Several evidences suggest that the position of palmitic acid (PA) in dietary triacylglycerol (TAG) influences different biological functions. We aimed at evaluating whether dietary fat with highly enriched (87%) PA in *sn*-2 position (H*sn*-2 PA), by increasing PA incorporation into tissue phospholipids (PL), modifies fatty acid profile and biosynthesis of fatty acid—derived bioactive lipids, such as endocannabinoids and their congeners.

**Study Design:**

Rats were fed for 5 weeks diets containing H*sn*-2 PA or fat with PA randomly distributed in TAG with 18.8% PA in *sn*-2 position (L*sn*-2 PA), and similar total PA concentration. Fatty acid profile in different lipid fractions, endocannabinoids and congeners were measured in intestine, liver, visceral adipose tissue, muscle and brain.

**Results:**

Rats on H*sn*-2 PA diet had lower levels of anandamide with concomitant increase of its congener palmitoylethanolamide and its precursor PA into visceral adipose tissue phospholipids. In addition, we found an increase of oleoylethanolamide, an avid PPAR alpha ligand, in liver, muscle and brain, associated to higher levels of its precursor oleic acid in liver and muscle, probably derived by elongation and further delta 9 desaturation of PA. Changes in endocannabinoids and congeners were associated to a decrease of circulating TNF alpha after LPS challenge, and to an improved feed efficiency.

**Conclusions:**

Dietary H*sn*-2 PA, by modifying endocannabinoids and congeners biosynthesis in different tissues may potentially concur in the physiological regulation of energy metabolism, brain function and body fat distribution.

## Introduction

The nutritional role of dietary palmitic acid (PA) is quite controversial. While it has been claimed to increase several risk factors, such as LDL cholesterol [1], inflammatory markers [2] and insulin resistance [3], when exceeding in the diet, it is also the major fatty acid in human milk and the main fatty acid produced endogenously. In fact, PA endogenous production guarantees its physiological steady membrane phospholipid (PL) concentration and the formation of oleic acid *via* elongation to stearic acid. Subsequent delta 9 desaturation yields oleic acid, which, after delta 6 desaturation, elongation and a further delta 5 desaturation, produces c20:3n9 [4, 5], called by the name who first discovered it, mead acid [6]. Mead acid, in case of essential fatty acid deficiency, replaces n-6 and n-3 20 carbon polyunsaturated fatty acids in membrane PLs [7], keeping membrane physical properties. PA in the PL form has also an important role as pulmonary surfactant [8]. In addition, PA esterified to retinol represent the major form of vitamin A storage in the liver [9] and via palmitoylation regulates several protein physiology and pathophysiology [10].

Moreover, PA also acts as precursor of molecules with high biological activities, such as palmitoylethanolamide (PEA). PEA possesses peculiar biological properties as antiinflammatory and analgesic agent [11], partially linked to its affinity to peroxisome proliferators activated receptor (PPAR) alpha and transient receptor potential cation channel (TRP) V1 [12], but likely also through alternative pathways [13]. In addition, also its metabolite oleic acid produces a N-acylethanolamide (NAE), oleoylethanolamide (OEA), a strong PPAR alpha agonist [14] with several biological activities in peripheral tissues [14–17] and brain [18, 19]. In order to be precursor of NAEs, fatty acids must be esterified in sn-1 position [20]. However, even though most of the PA in the body is esterified in sn-1 of PLs, it is not known whether dietary PA influences PEA biosynthesis. In vitro studies in mouse adipocytes seem to confirm this possibility [21]. On the other hand, it is not known whether the distribution of PA in dietary triglycerides, by influencing its incorporation into PLs is able to influence endocannabinoids and congeners biosynthesis. It has been shown that the position of PA in the triacylglycerol (TAG) backbone influences several nutritional activities, in particular in infants [22]; among them the effects on food intake and weight gain may be re-conducted to the modulation of endocannabinoid and congeners biosynthesis in different tissues [23].

Interestingly, some of the nutritional properties of human milk fat, a high sn-2 PA fat (75%) have been attributed to PA in position *sn*-2 [22].

In this paper, we aim at verifying whether a high concentration of sn-2 PA affects PA incorporation into PLs and influences endocannabinoids and congeners biosynthesis. In addition since, as mentioned above, PA has been claimed to possess pro-inflammatory activity [2], we also evaluated, after treatment with a single dose of LPS, whether its esterification in sn-2 position influences the susceptibility to inflammation.

## Materials and Methods

### Animals and treatments

40 male wistar rats weight 100–150g were housed for a week before randomly allocating to two diets 10% high *sn*-2 PA fat based diet (Hsn-2 PA) and 10% low sn-2 PA fat based diet (Lsn-2 PA). High and low sn-2 PA fat diets had similar total PA content (see **Table 1** for the diet fatty acid composition).

**Table 1:** Fatty acid composition, as % of total fatty acids, of the dietary fat with high in palmitic acid in sn-2 position (Hsn-2 PA) or with low in palmitic acid in sn-2 position (Lsn-2 PA).

	Hsn-2 PA	Lsn-2 PA
	% of total fatty acids	
Palmitic in sn-2	87.1	18.8
c14:0	1.3	3.4
c16:0	23.5	24.2
c16:1	2.1	3.4
c18:1	40.3	37.8
c18:2	30.2	28.4
c18:3	0.7	0.4

Rats were fed for 5 weeks. Weight of the animals and length were recorded weekly. Food intake was recorded every 2 days.

In order to evaluate the response to an inflammatory stimulus, 10 animals per group were randomly assigned for the treatment 12h before sacrifice with i.p. LPS 0.5mg/Kg of body weight.

Before sacrifice, rats were fasted for 12h, and killed by decapitation. Adipose tissue, small intestine, liver, muscle and brain were taken and processed for lipid analyses. Plasma was separated from blood and processed for cytokines analyses.

All experiments were performed according to the guidelines and protocols approved by the European Union (EU Council 86/609; D.L. 27.01.1992, no. 116) and by the Animal Research Ethics Committee of the University of Cagliari, Italy.

### Cytokine measurements

TNFα, IL-1 and IL-6 were determined as previously reported [24]. Briefly, cells from whole blood were removed by centrifugation of 400 g for 10 min and supernatants frozen at −20°C until evaluation. Cytokines assay was performed with a sandwich ELISA test (Biosource, Nivelles, Belgium). Absorbance at 450 nm was measured with a model 680 microplate reader (Bio-rad, Hercules, CA). A standard curve was prepared by plotting absorbance value of the standard cytokines versus the corresponding concentration (pg/mL or ng/mL). Cell protein content was measured by Bradford assay [25].

### Lipid analysis

Total lipids were extracted by the method of Folch [26]. Separation of lipid fractions, PL, TAG and non esterified fatty acids (NEFA), from total lipids was performed as previously reported [27]. In brain tissue the extracted TAG and NEFA fractions resulted not sufficient for fatty acid measurement.

Aliquots were mildly saponified as previously described [28] in order to obtain free fatty acids for HPLC analysis. Separation of unsaturated fatty acids was carried out with an Agilent 1100 HPLC system (Agilent, Palo Alto, Calif., USA) equipped with a diode array detector as previously reported [29]. N-acylethanolamides and 2-acylglycerols were measured as previously described [30]. Since saturated fatty acids are transparent to UV detection, they were measured after methylation as depicted in [31], of free fatty acids obtained as described above, by gas chromatography as described in [32].

### Statistical analyses

Statistical differences between the two experimental groups was evaluated with t-student test where P values <0.05 were considered significant.

## Results

**Fig 1:**
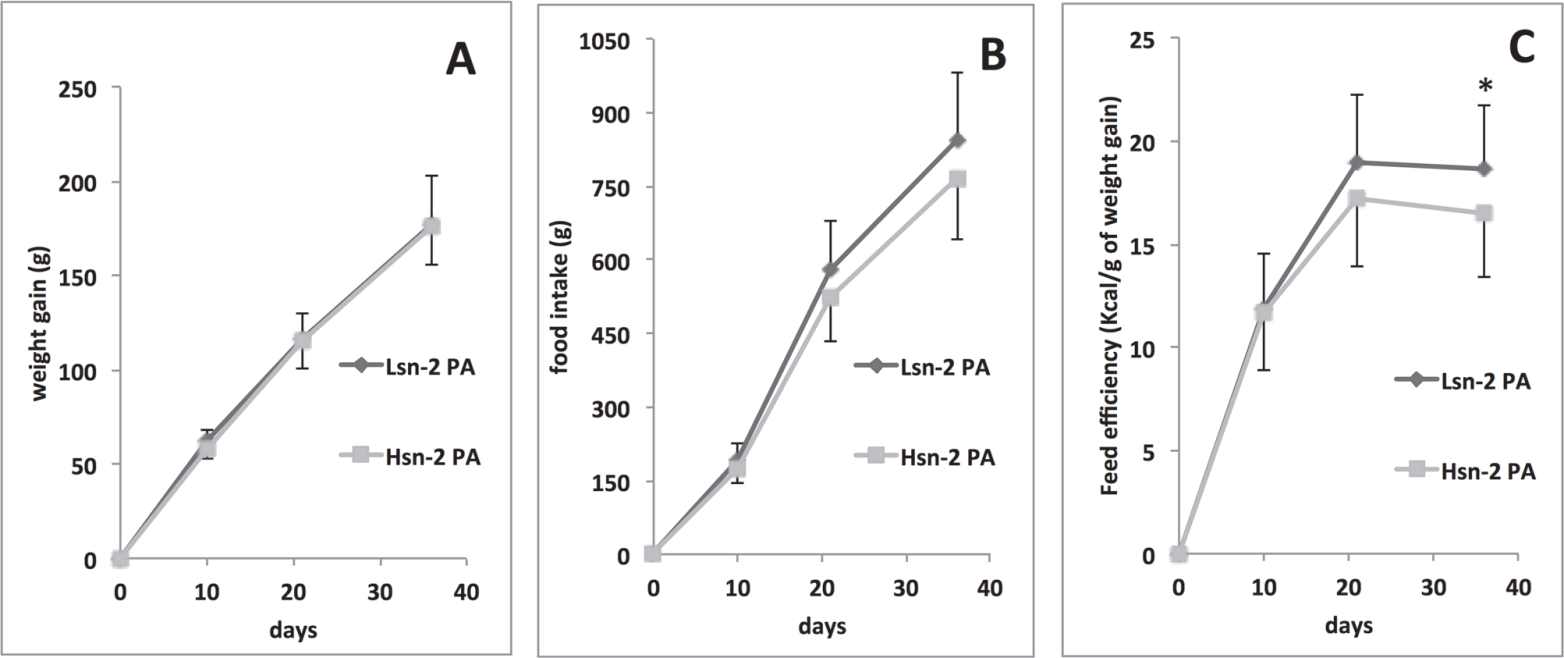
Weight gain (A), food intake (B) and feed efficiency (C) in rats fed Hsn-2 PA or Lsn-2 PA diet for 5 weeks. Feed efficiency is calculated as the amount of Kcal intake needed to gain 1 g of body weight. Therefore, to lower values correspond better feed efficiency. Weight of the animals was recorded weekly, food intake was recorded every 2 days. Values are means ± SD, n = 20. * P< 0.05.

As shown in **Fig. 1**, feeding H*sn*-2 PA diet resulted in a significantly higher feed efficiency with a lower food intake even though not significant, with respect to rats fed the Lsn-2 PA.

In small intestine, compared to L*sn*-2 PA, H*sn*-2 PA diet statistically increased PA in PLs (**Table 2**), which was associated to an increase of 2-palmitoyl monoacylglycerol (2-PG) (**Table 2**).

**Table 2:** Small intestine N-Acylethanolamides (NAEs), 2-acylglycerols (2-AcylGs) and fatty acid concentrations in phospholipids (PL), triacylglycerols (TAG) and non esterified fatty acids (NEFA), in rats fed Lsn-2 PA or Hsn-2 PA for 5 weeks.

	Lsn-2 PA	Hsn-2 PA
	NAEs and 2-AcylGs (pmoles/g of tissue)	
AEA	0.49 ± 0.04	0.41 ± 0.09
PEA	0.36 ± 0.08	0.27 ± 0.05
OEA	1.47 ± 0.39	1.07 ± 0.68
2-AG	37.37 ± 5.23	38.83 ± 6.87
2-PG	1.48 ± 0.40	2.21 ± 0.21*
2-OG	2.98 ± 1.21	1.90 ± 0.92
	PL fatty acids (umoles/g of tissue)	
14:0	3.56 ± 2.07	3.27 ± 0.89
16:0	32.13 ± 8.62	49.72 ± 9.15*
18:0	12.36 ± 2.44	10.47 ± 2.95
16:1n-7	7.12 ± 3.75	7.55 ± 2.47
18:1n-9	41.63 ± 22.19	41.08 ± 14.55
18:2n-6	10.66 ± 4.37	9.03 ± 3.06
20:4n-6	6.07 ± 1.08	4.98 ± 2.17
22:6n-3	0.69 ± 0.15	0.55 ± 0.24
	TAG fatty acids (nmoles/g of tissue)	
16:0	415.48 ± 175.35	520.57 ± 219.37
18:0	75.31 ± 32.49	67.79 ± 28.41
18:1n-9	529.81 ± 102.47	502.82 ± 110.43
18:2n-6	310.51 ± 131.49	328.91 ± 141.38
	NEFA fatty acids (nmoles/g of tissue)	
16:0	60.28 ± 26.57	72.35 ± 31.56
18:0	12.32 ± 5.23	15.85 ± 2.96
18:1n-9	83.52 ± 31.11	101.48 ± 37.59
18:2n-6	34.39 ± 11.78	42.63 ± 15.75

AEA, anandamide; PEA, palmitoylethanolamide; OEA, oleoylethanolamide; 2-AG, 2 arachidonoylglycerol; 2-PG, 2 palmitoylglycerol; 2-OG, 2-oleoylglycerol. Values are means ± SD, n = 10.

* P< 0.05.

Also in visceral adipose tissue PLs, a statistically significant higher PA concentration was detected when rats were fed the H*sn*-2 PA diet with respect to those fed L*sn*-2 PA diet (**Table 3**). This difference in concentration was associated to an increase of palmitoylethanolamide (PEA) and a decrease of anandamide (AEA) (**Table 3**).

**Table 3:** Visceral adipose tissue N-Acylethanolamides (NAEs), 2-AG and fatty acid concentrations in phospholipids (PL), triacylglycerols (TAG) and non esterified fatty acids (NEFA), in rats fed Lsn-2 PA or Hsn-2 PA for 5 weeks.

	Ln2-PA	Hsn2-PA
	NAEs and 2-AG (pmoles/g of tissue)	
AEA	49.64 ± 20.80	22.32 ± 4.69*
PEA	439.15 ± 153.64	632.18 ± 89.77*
OEA	646.73 ± 185.72	592.30 ± 192.45
2AG	447.35 ± 211.08	506.87 ± 163.49
	PL fatty acids (umoles/g of tissue)	
14:0	0.52 ± 0.05	0.45 ± 0.07
16:0	5.74 ± 0.68	6.83 ± 0.47*
18:0	1.53 ± 0.18	1.60 ± 0.13
16:1n-7	2.63 ± 0.47	3.15 ± 1.00
18:1n-9	15.16 ± 1.22	17.47 ± 3.77
18:2n-6	3.13 ± 0.45	3.49 ± 0.67
20:4n-6	0.85 ± 0.08	0.85 ± 0.16
18:3n-3	0.17 ± 0.05	0.21 ± 0.04
22:6n-3	0.06 ± 0.01	0.06 ± 0.01
	TAG fatty acids (umoles/g of tissue)	
14:0	101.21 ± 4.98	97.78 ± 5.53
16:0	841.94 ± 45.58	927.81 ± 85.95
18:0	109.93 ± 2.45	110.28 ± 5.54
18:1n-9	1053.73 ± 97.51	1063.59 ± 88.28
18:2n-6	190.67 ± 23.90	191.16 ± 17.23
	NEFA fatty acids (nmoles/g of tissue)	
16:0	3.25 ± 1.71	3.91 ± 1.50
18:0	0.68 ± 0.25	0.57 ± 0.20
18:1n-9	4.89 ± 2.10	4.32 ± 1.75
18:2n-6	1.54 ± 0.39	1.29 ± 0.53

AEA, anandamide; PEA, palmitoylethanolamide; OEA, oleoylethanolamide; 2-AG, 2-arachidonoylglycerol. Values are means ± SD, n = 10.

* P< 0.05.

The increase of PA in PLs was also found in the liver of rats fed H*sn*-2 PA diet with respect to those fed L*sn*-2 PA diet (**Table 4**). Interestingly, in the liver was found an increase of OEA (**Table 4**) after H*sn*-2 PA feeding, while the other amides and 2-AG did not change significantly.

**Table 4:** Liver N-Acylethanolamides (NAEs), 2-AG, and fatty acid concentrations in phospholipids (PL), triacylglycerols (TAG) and non esterified fatty acids (NEFA), in rats fed Lsn-2 PA or Hsn-2 PA for 5 weeks.

	Ln2-PA	Hsn2-PA
	NAEs and 2-AG (pmoles/g of tissue)	
AEA	37.76 ± 17.13	26.86 ± 1.67
PEA	188.58 ± 97.44	194.84 ± 27.12
OEA	305.76 ± 53.81	521.83 ± 12.81*
2-AG	11042.08 ± 3100.20	9562.52 ± 1414.53
	PL fatty acids umoles/g of tissue)	
16:0	17.56 ± 1.36	19.63 ± 0.52*
18:0	21.25 ± 1.79	21.80 ± 2.04
18:1n-9	5.82 ± 0.30	6.02 ± 0.31
18:2n-6	8.37 ± 0.62	8.08 ± 0.61
20:4n-6	17.75 ± 1.67	18.50 ± 0.42
22:6n-3	4.68 ± 0.38	4.92 ± 0.31
	TAG fatty acids (umoles/g of tissue)	
16:0	11.73 ± 2.96	11.96 ± 1.40
18:0	1.66 ± 0.20	1.79 ± 0.07
16:1n-7	1.32 ± 0.47	1.45 ± 0.22
18:1n-9	10.02 ± 3.39	10.39 ± 2.06
18:2n-6	2.79 ± 0.77	2.69 ± 0.56
	NEFA fatty acids (nmoles/g of tissue)	
16:0	145.33 ± 44.83	175.44 ± 45.29
18:0	20.62 ± 6.35	16.28 ± 5.95
18:1n-9	142.83 ± 47.48	209.98 ± 62.58
18:2n-6	66.40 ± 19.88	56.23 ± 12.95
20:4n-6	77.21 ± 20.93	55.97 ± 21.05
22:6n-3	28.46 ± 11.16	33.93 ± 14.34

AEA, anandamide; PEA, palmitoylethanolamide; OEA, oleoylethanolamide; 2-AG, 2-arachidonoylglycerol; Values are means ± SD, n = 10.

* P< 0.05.

The results in muscle tissue reflected those found in the liver, with a significant increase of PA in PLs and of OEA (**Table 5**) in rats fed H*sn*-2 PA compared to those fed Lsn-2 PA.

**Table 5:** Muscle N-Acylethanolamides (NAEs), 2-AG and fatty acid concentrations in phospholipids (PL), triacylglycerols (TAG) and non esterified fatty acids (NEFA), in rats fed Lsn-2 PA or Hsn-2 PA for 5 weeks.

	Ln2-PA	Hsn2-PA
	NAEs and 2-AG (pmoles/g of tissue)	
AEA	17.50 ± 4.74	15.96 ± 3.44
PEA	105.46 ± 34.62	86.32 ± 8.51
OEA	313.66 ± 11.42	400.95 ± 55.54*
2AG	689.34 ± 35.09	771.44 ± 134.88
	PL fatty acids (umoles/g of tissue)	
16:0	7.51 ± 0.31	8.97 ± 1.02
18:0	4.88 ± 0.14	5.37 ± 0.79
16:1n-7	0.35 ± 0.01	0.44 ± 0.02
18:1n-9	2.47 ± 0.16	2.72 ± 0.21
18:2n-6	5.55 ± 0.31	5.43 ± 0.30
20:4n-6	3.15 ± 0.33	3.23 ± 0.04
22:6n-3	2.48 ± 0.13	2.88 ± 0.22
	TAG fatty acids (nmoles/g of tissue)	
14:0	259.09 ± 89.57	157.15 ± 71.04
16:0	2712.80 ± 695.19	2276.43 ± 804.75
18:0	718.05 ± 144.67	622.58 ± 106.40
16:1n-7	58.54 ± 13.76	41.99 ± 28.77
18:1n-9	297.62 ± 119.62	207.37 ± 90.59
18:2n-6	63.60 ± 33.57	41.21 ± 16.49
	NEFA fatty acids (nmoles/g of tissue)	
16:0	150.68 ± 40.56	185.89 ± 35.87
18:1n-9	145.51 ± 32.12	203.92 ± 20.85*
18:2n-6	42.05 ± 6.31	45.59 ± 4.99
20:4n-6	15.15 ± 1.31	16.11 ± 1.93
22:6n-3	29.43 ± 2.84	35.01 ± 4.39

AEA, anandamide; PEA, palmitoylethanolamide; OEA, oleoylethanolamide; 2-AG, 2-arachidonoylglycerol; Values are means ± SD, n = 10.

* P< 0.05.

In addition, in the muscle of H*sn*-2 PA fed rats we found a significant increase of oleic acid (OA) but only in the non esterified fatty acid (NEFA) fraction (**Table 5**).

Analysis of brain tissue showed an increase of OEA (**Table 6**).

**Table 6:** Brain N-Acylethanolamides (NAEs), 2-AG, and phospholipid (PL) fatty acid concentrations, in rats fed Lsn-2 PA or Hsn-2 PA for 5 weeks.

	Ln2-PA	Hsn2-PA
	NAEs and 2-AG (pmoles/g of tissue)	
AEA	54.80 ± 8.29	57.71 ± 8.03
PEA	427.91 ± 35.62	373.14 ± 60.72
OEA	546.95 ± 115.32	767.62 ± 100.20*
2AG	15126.88 ± 4020.74	14166.69 ± 3857.84
	PL fatty acids (umoles/g of tissue)	
16:0	30.33 ± 0.33	30.82 ± 0.77
18:0	22.22 ± 0.82	22.14 ± 0.50
18:1n-9	22.30 ± 3.71	24.14 ± 4.53
18:2n-6	0.57 ± 0.10	0.60 ± 0.10
20:4n-6	10.39 ± 1.01	11.99 ± 1.63
22:4n-6	2.12 ± 0.29	2.48 ± 0.44
22:6n-3	11.51 ± 0.86	12.71 ± 1.12

AEA, anandamide; PEA, palmitoylethanolamide; OEA, oleoylethanolamide; 2-AG, 2-arachidonoylglycerol; Values are means ± SD, n = 10.

* P< 0.05.

In the plasma of rats fed Hsn-2 PA diet, we found lower levels of TNF alpha and IL-1, even though the latter not statistically significant, with respect to those fed Lsn-2 PA diet (**Table 7**), after treatment with a single dose of LPS.

**Table 7:** Plasma TNF-alpha, IL-1 and IL-6 concentrations in rats fed Lsn-2 PA or Hsn-2 PA for 5 weeks and treated 12h before sacrifice with i.p. single dose of LPS (0.5mg/Kg of body weight).

	Hsn-2 PA	Hsn-2 PA
	pg/ml plasma	
TNF-alpha	13.36 ± 3.35	9.94 ± 1.65*
IL-1	508.84 ± 88.07	460.54 ± 47.12
IL-6	107.51 ± 11.09	98.04 ± 19.21

Values are means ± SD, n = 10.

* P< 0.05.

## Discussion

Our data show that the position of dietary PA in sn-2 of TAGs results in a better incorporation of PA in intestine, adipose tissue and liver PLs. Interestingly, the increase of PA was associated to an increase of PEA and decrease of AEA in adipose tissue, while higher levels of OEA were found in all other tissues tested. It seems therefore that PA increase in PLs is able to modify NAEs profile in different tissues. PEA and AEA have different activities in different tissues and experimental conditions. For instance, in visceral adipose tissue PEA has been shown to possess antiinflammatory properties [33], while AEA, acting as CB1 and PPAR gamma ligand, promotes adipogenesis [34]. Therefore, higher production of PEA and lower of AEA in visceral adipose tissue, as found in rats fed Hsn-2 PA diet, may reduce inflammatory state and adipogenesis. The lower deposition of fat in visceral adipose tissue, might favor fat accumulation in subcutaneous adipose tissue. Interestingly, pig fat is characterized by a high portion of PA in sn-2 of the TAG backbone, and it has been shown that subcutaneous porcine preadipocytes proliferated more actively and showed more rapid accumulation of TAG than visceral derived adipocytes [35]. Similarly, human milk contains most of PA in sn-2 position, and babies accumulate fat mainly in the subcutanoeus compartments [36]. It is well known that accumulation in subcutaneous adipose tissue does not lead to dismetabolism and insulin resistance as opposite to an excess accumulation in visceral adipose tissue, which leads to visceral obesity [37]. Interestingly, several studies demonstrated the protective effect of breastfeeding against obesity in childhood [38, 39] and adulthood [40–43]. Future studies will aim at verifying whether Hsn-2 PA may be a nutritional tool to significantly affect body fat distribution and thereby to protect from visceral obesity.

The increase of PEA in adipose tissue was associated to a reduction of plasma TNF alpha after treatment with LPS, while other pro-inflammatory cytokines such as IL-1 and IL-6 did not change significantly. Whether the effect of Hsn-2 PA is limited to TNF alpha, as it has been shown for PEA [13], or the anti-inflammatory activity is not sufficiently strong to also influence IL-1 and IL-6 levels, remains to be elucidated.

Peculiarly, in the other tissues we found increased levels of OEA and not PEA as one could expect. We may hypothesized that in the liver PA may undergo elongation and delta 9 desaturation to form OA and thereby OEA. However, we did not find any change in OA, only in muscle we found a significant higher level of OA in the NEFA fraction. Interestingly, recently it has been shown that dietary Hsn-2 PA increases non esterified plasma 18:0 in term infants [44], which may enter into muscle and, after delta 9 desaturation, form OA.

On the other hand, the no changes of OA found in other lipid fraction, is likely due to the abundant level of oleic acid in all tissues. In addition, in vitro studies in rat horizontal slices containing the midbrain, incubated for 1h with PA [45], we found increased levels of OEA but not of PEA and no change in PA concentration.

The global increase of OEA in different tissues may have important implications in terms of energy metabolism and body composition. In fact, OEA has been demonstrated to be a strong ligand of PPAR alpha, which regulates fatty acid metabolism in liver and muscle [46] and modulate several functions in the CNS [45]. Recently, it has been shown that OEA, is able to regulate dopamine homeostasis [47], consolidate memory [18], and may also influence hunger/satiety circuitry [48].

This could also explain the lower food intake, albeit not significant, recorded in Hsn-2 PA fed rats even though did not result in changes in weight gain but in an improved feed efficiency. Interestingly, breast fed babies has been shown to have lower food intake with respect to those fed infant formula [49], while whether they have similar growth is still debated [50]. Targeted experiments should be carried out in order to evaluate the possible effect of Hsn-2 PA in favoring a physiological growth.

To our knowledge, this the first report showing an influence of the dietary fatty acid position in the TAG backbone on endocannabinoid and congeners biosynthesis. Based on our data, the fatty acid in sn-2 position from the diet is preferentially incorporated into tissue PLs. Recently, it has been shown that dietary Hsn-2 PA increases PA in sn-2 TAG in term infants plasma [44]. Whether the preferential incorporation of PA into sn-2 plasma TAG favors tissue incorporation of PA into PLs should be further investigated.

There is at least one more example on the influence of the different properties of dietary fatty acids based on their form, on endocannabinoid biosynthesis. It has been shown that dietary EPA and DHA in PL form down-regulate endocannabinoid biosynthesis more efficiently than in the dietary TAG form, in experimental animals [32] and humans [51].

Overall our data demonstrate that Hsn-2 PA by increasing PA in tissue PLs modulate NAEs biosynthesis in different tissues resulting in a lower susceptibility to inflammation, improved feed efficiency, and opens to possible activities in the regulation of fat deposition and brain function.
